# Research into the efficacy and cost-effectiveness of brief, free of charge and anonymous sex counselling to improve (mental) health in youth: Design of a randomised controlled trial

**DOI:** 10.1186/1471-2458-9-459

**Published:** 2009-12-13

**Authors:** Andrea Grauvogl, Silvia MAA Evers, Katy van den Hoek, Evert van der Veen, Anja Franke, Jacques JDM van Lankveld

**Affiliations:** 1 Department of Clinical Psychological Science, Faculty of psychology, Maastricht University, the Netherlands; 2 Department of Health, Organization, Policy and Economics, Faculty of Health, Medicine and Life Sciences, Caphri, Maastricht University, the Netherlands; 3 Infectious diseases, GGD Rotterdam-Rijnmond, the Netherlands

## Abstract

**Background:**

The capacity to form romantic relationships and sexual health of adolescents in the Netherlands are compromised by several factors, including young age of first intercourse and adolescent depression. Several thresholds like own expenses, trust and embarrassment prevent adolescents to seek help for their sexual problems. To overcome these thresholds, brief sex counselling has been developed. It has been used since 2006 within the Rotterdam-Rijnmond Public Health Service, but there is lack of information about the (cost-) effectiveness. In the current study we will evaluate the (cost-) effectiveness of brief sex counselling for sexual problems in adolescents and young adults between 18 and 25 years of age.

**Methods:**

In a randomised controlled trial we will compare (1) brief sex counselling with (2) intensive sexological treatment, and (3) delayed treatment (waiting list). Embedded in this RCT will be a trial-based economic evaluation, looking at the cost-effectiveness and cost-utility of brief sex counselling versus the two other interventions. Four hundred fifty adolescents (aged 18-25) with sexual problems will be recruited among the persons who visit the Public Health Service (PHS) and through various websites. After a screening procedure, eligible participants will be randomly allocated to one of the three intervention groups. Primary outcome measure of the clinical evaluation is the severity of sexual problems. Other outcomes include psychological distress, especially depression. The economic evaluation will be performed from a societal perspective. Costs will be assessed continuously by a retrospective questionnaire covering the last 3 month. All outcome assessments (including those for the economic evaluation) will take place via the internet at baseline, and at 3, 6, 9, and 12 months after baseline.

**Discussion:**

The proposed research project will be the first study to provide preliminary data about the effect and cost-effectiveness of brief sex counselling in youth in comparison with intensive sexological treatment and delayed treatment. It is anticipated that positive results in (cost-) effectiveness of the proposed intervention will contribute to the improvement of sexual health care for adolescents and young adults.

**Trial registration:**

The study has been registered at the Netherlands Trial Register, part of the Dutch Cochrane Centre (NTR1952)

## Background

Sexual health of adolescents has gained more attention over the last years [1,2]. Sexual health is described by the World Health Organisation (WHO) as:

"... a state of physical, emotional, mental and social well-being in relation to sexuality; it is not merely the absence of disease, dysfunction or infirmity. Sexual health requires a positive and respectful approach to sexuality and sexual relationships, as well as the possibility of having pleasurable and safe sexual experiences, free of coercion, discrimination and violence. For sexual health to be attained and maintained, the sexual rights of all persons must be respected, protected and fulfilled [3]".

The past decade has also seen a growing recognition of the existence of romantic relationships, experiences and sexual activities in adolescents [4]. With this attention some developments have taken place that could compromise the sexual health and romantic relationship capacities of adolescents.

The first development is the increasing proportion of young people that have sexual intercourse before the age of 16 [5]. Sexual intercourse before this age is associated with increased risks [6]. Men and women who had their first sexual encounter before the age of 16 show increased regret of this experience [5,7,8]. For both, boys and girls, the early onset of intercourse was associated with the experienced pressure by peers, and in girls this was also associated with the lack of control in the process of and during sexual intercourse [5]. Another study found increased rates of coercion to have sex in women who had their first sexual intercourse at a younger age. It is also found that the incidence of sexually transmitted diseases (STD's) was higher for the group who had their first sexual intercourse before the age of 16 [6,7]. Girls who began sexual activity before the age of 13 were twice as likely to become infected with an STD as girls who started sexual activity after the age of 21. Other associations of early sexual activity were a greater likelihood of becoming a single mother, more abortions, and lower levels of personal happiness [6].

Furthermore the younger a person was at his/her first sexual intercourse the higher the likelihood was of having multiple sex partners (at least five sex partners lifetime or four sex partners during the last six months). Having multiple sex partners is associated with (sexual) risk behaviours such as unsafe sex (not using contraceptives), smoking, drug- and alcohol use [8-11].

As mentioned before, unsafe sex is a common risk factor among adolescents and young adults. It has been found that only in the US, in 19 million new STD cases that occur every year, half of these are in individuals between 15 and 24 years of age [12]. Pregnancy is of course another consequence of unsafe sex. Darroch, Frost and Singh (2001) found that the US has the highest pregnancy and birth rate in women between 15 and 19 years compared to other developed countries. These higher rates arise primarily because of less, and probably less effective contraceptive use [13].

It is well known that mood disorders, and especially major depressive disorder, are among the most common mental disorders in adolescence [14-16]. Mental disorders, including depression, play a role in the development of sexual risk behaviour and sexual health problems [17,18]. Sex- and drugs-related behaviour predicts an increased likelihood of depression. Among girls, both experimental behaviour patterns (experimenting with sex, drugs and alcohol) and high-risk behaviour patterns (excessive drinking and/or drug use, and unprotected sexual contact) were found to increase the odds of subsequent depression. Among boys only high-risk behaviour patterns increased the odds of the development of subsequent depression [19].

Romantic attractions, activities (dating, kissing, more than kissing (like touching each other), etcetera) and relationships were also associated with a greater risk of depressive symptoms in adolescence [4,20]. Adolescents who had romantic relationships and who engaged in romantic activities, compared with adolescents who had not, reported increased depressive symptoms over time [21]. Several models have been proposed to explain this association. For example, the attention impairment model [22], suggests that romantic activities takes attention away from other important areas of functioning and so causing impairment, which increases the risk for depressive symptoms. Impairment of romantic functioning (interpersonal skills in the romantic domain) impaired by depressive symptoms can furthermore have far reaching consequences for the relational development of adolescents [4].

Another study in 7^th^-12^th ^grade students, age 13 to 18, found that depressive symptoms were associated with increased sexual risk. Among boys depressive symptoms were associated with non condom use at last sexual intercourse, among both boys and girls it was associated with a history of STD [17,23]. Thus, the relationship in adolescents and young adults between sexual behaviour and sexual risk behaviour on the one hand, and psychopathology, particularly depression, on the other hand, appears to be bidirectional.

In the Netherlands, adolescents are - in general - sexually healthy but there are some alarming developments. Bakker, et al. (2009) examined the sexual health of 6428 men and women between 15 and 70 years old (girls 15-18 years old N = 443; boys 15-18 years old N = 368). Of the 15 and 16 year olds, 33% of the girls and 25% of the boys had sexual intercourse. At the age of 17-18 these percentages have almost doubled. Girls with a lower education level have significantly more sexual experience than higher educated girls. Of the 15 to 18 year olds, 3.8% of the boys, and 2.9% of the girls have had more than 10 sexual partners up to that point of their lives. Furthermore, 32.6% of these boys and 28.5% of these girls experienced feelings of guilt about their sexual behaviour. Pregnancy and birth control were also examined in this study. Among boys, 1.1% of their female sexual partners had been pregnant and 0.4% had the baby. More of the partners of high-educated men under 26 years of age who experienced unwanted pregnancy had abortions than low educated men, but the partners of the latter more often gave childbirth. Among girls, 3.7% had been pregnant and 0.8% had the baby. Low educated girls under 26 years of age had more (unexpected) pregnancies, children, and abortions than high educated girls. Among boys, 58.9% uses no contraceptives, while this is the case for 40.9% of the girls. Sexual victimization is more common among girls than among boys (20.2% vs. 2.3%) [24]. Comparable results have been found in other studies in the Netherlands [1,25].

To improve this situation the Dutch national government has created a new service that facilitates assistance in the area of young peoples' sexual health. The core is a nationwide network of sexual health service provision centres (called 'Sense') for young people up to 25 years of age with questions concerning or problems with sexual health. The regulation builds on the experiences of the pilot version of Sense (pilot from 2005-2008), which was situated in three areas. One of them being the southern South-Holland (sSH) region. In the pilot version of Sense sSH, the Rotterdam-Rijnmond Public Health Service (PHS) identified an omission. Referral of adolescents with sexual health problems could not be performed adequately; the threshold for referral is too high (for example too expensive, too far away, etcetera). Research has shown that not everyone who needed help or advice for sexual health issues actually sought or had access to that help. The unfamiliarity with the available resources is one factor. Furthermore, the lack of trust in the available help, the costs, and reluctance of the help-seeking person are factors that play a role [24]. This implies that a large group of young people who need help for sexual issues have no access to the necessary care. For this reason the Rotterdam-Rijnmond PHS has, in addition to Sense, appointed a sex counsellor who helps to address the sexual problem in a maximum of three, free of charge and anonymous consultations of 45 minutes maximum. This service is called brief sex counselling. Its aim is to provide tailored and practical advice and/or reference to specialised health care, and to bridge the gap between Sense and this, more intensive, specialized help.

The earlier mentioned developments [24] regarding sexual health and the related mental health problems [17,18] form a major problem for youth in the Netherlands. Brief sex counselling might be a solution as it aims to treat the sexual problems of these adolescents in an early stage. Related mental disorders (like depression) would be less likely to develop or escalate. It is expected that brief sex counselling will prevent more serious and chronic problems in the future. However, there is no scientific evidence yet.

Goldmeier and colleagues calculated that, in 2004, the average costs of the treatment of sexual dysfunctions in the UK for women were €417 when delivered by a psychologist and €896 when delivered by a sex therapist [26]. Potentially, a certain proportion of patients with sexual problems do not require this type of intensive and costly treatment, but can adequately and satisfactorily be treated with less intensive and lower-cost alternatives. For instance, in the Netherlands it was estimated that 14% (respectively 56,000 of the extrapolated total of 400,547) of the patients treated in hospital outpatient clinics for sexology and 24% (respectively 80,500 of the extrapolated total of 335,594) of the patients treated in mental health care centres, could be adequately treated with a cheaper, low-threshold intervention [25,27]. It can be expected that brief sex counselling is a cheaper and more approachable option to treat sexual problems in adolescents than standard sexual health care provided in community mental health services or hospital outpatient clinics for sexology.

There are two main reasons to conduct this study that are related to current policy aims to improve the sexual health in youth. First, it can be assumed that brief sex counselling for high risk groups has a preventive impact on the recurrence of their sexual condition. This would reduce, moreover, the patient flow to more expensive health care in hospitals and mental health care institutions [25]. Second, a secondary prevention effect of sex counselling on mental health problems, in particular depression, is predicted [25,28].

The objectives of the current study are threefold. The first is the development of protocols for brief sex counselling in the field of sexual health to address problems in sexual functioning in youth. Furthermore a training plan will be developed to train nurses and public health workers to implement these protocols for brief sex counselling. The second objective is to investigate the effects of brief sex counselling on somatic, mental and sexual health. The third and last aim is to investigate the cost-effectiveness of brief sex counselling. We will examine whether brief sex counselling is preferable to more intensive formats of sexual health care and delayed treatment in terms of costs, impact and utilities from a social perspective.

## Methods

### Design

**Figure 1:**
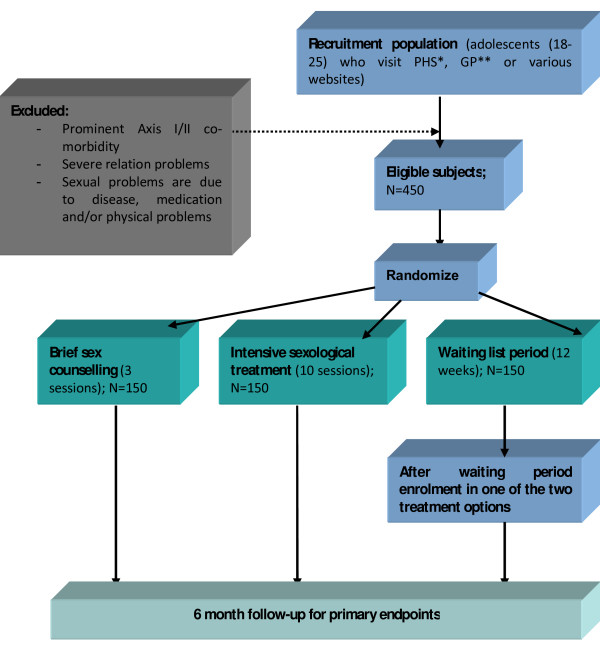
**Trial flow proposed participants enrolment and randomization procedures**. PHS*: Public Health Service, GP**: General Practitioner.

The proposed study is a prospective, randomised controlled trial (RCT). The subjects will be randomized to one of the three conditions: (1) brief sex counselling, (2) intensive sexological treatment, and (3) a delayed treatment (waiting list). The trial flow of the proposed subject enrolment and randomization procedures are graphically shown in Figure 1. This study has been approved by the ethics committee academic hospital Maastricht/Maastricht University, the Netherlands. Embedded in this RCT will be a trial-based economic evaluation to investigate the cost-effectiveness and cost-utility of brief sex counselling versus intensive sexological treatment (*see *Figure 1).

### Participants

The patient population we aim to investigate consists of adolescents with sexual problems. Patients are eligible to participate if they meet the following criteria: age between 18 and 25 years old; at least mild to moderate sexual problems (based on Questionnaire for the Screening of Sexual Dysfunction [29,30], International Index of Erectile Function (men) [31] and Female Sexual Functioning Index (female) [32]); no severe psychiatric co-morbidity or relational problems, and the sexual problems are not caused by disease, medication use or physical disabilities.

### Interventions

#### Brief sex counselling

Brief sex counselling is a brief, free of charge and easily accessible treatment for sexual problems of people between 18 and 25 years of age. In a maximum of three sessions, of 45 minutes each, a sex counsellor attempts to solve the problems or refers to more specialized help. The treatment is aimed at people without relationship and/or psychiatric problems. The sessions include providing information, psycho-education and limited advice on behaviour change. Furthermore home exercise assignments can be given, and the experiences with these exercises are discussed. The possibility for adolescents to talk about their sexuality in a non-threatening environment could initiate the problem-solving process.

#### Intensive sexological treatment

In a maximum of 10 treatment sessions a (Dutch Association for Sexology) certified sexologist offers an intensive form of treatment for the sexual problem(s) of the participant. This is the standard format of care for adults with sexual problems. In contrast to brief sex counselling, intensive sexological treatment also addresses the relational and/or personal psychological problems which possibly underlie the base of the sexual problems. The treatment is largely based on cognitive-behavioural therapy. In many cases homework assignments have to be carried out. The participant is taught to recognize and change thoughts which could play a negative role in their sex life.

#### Delayed treatment

In the event of the participant being randomized into the waiting list, a period of 26 weeks follows in which no treatment is offered. Participants are requested not to engage in other types of professional treatment. At the end of the waiting list period the participant can choose between brief sex counselling and the intensive sexological treatment.

### Protocol

The protocol that has been developed for the brief sex counselling sessions is based on the principles and practice of sex counselling in general and on a guideline that was developed for the Sense sessions [33,34]. The protocol chapters address problems of sexual desire, sexual arousal, orgasm and sexual pain. First, a description of the disorder is given. Next, specific questions are provided for sexual history taking. Furthermore an action plan is provided. Most of the time is spent on the action plan. This describes what has to be or can be done to solve the problem. The first step in the plan is providing information. This is done to improve the client's knowledge about the cause of his or her problem and the factors that maintain it. A second action point concerns cognitive and behavioural aspects. Several compromised cognitive processes and deficient coping strategies can underlie the sexual problems. During the sessions an attempt is made to identify, change and adjusted these processes and strategies. The protocol provides examples of these processes and strategies and how to deal with them. The last action point concerns teaching of sensate-focus exercises. These exercises aim to help a person or couple to (re)discover sexual needs and preferences. Different exercises provided are tailored to address different sexual problems.

### Research Questions

Both for the effectiveness study as well as for the economic evaluation study we have defined a number of research questions:

#### Effectiveness study

1. Does brief sex counseling as envisaged, when compared to delayed treatment, improve the sexual and general mental health of adolescents and young adults? To which extent (effect size) and in what respect (which outcome dimensions)?

2. Is brief sex counseling efficacious, when compared to delayed treatment, in preventing escalation of (sexual and mental) health problems compared with no help?

#### Economic evaluation

1. Is, from a social perspective, the delivery of brief sex counselling compared to intensive sexological treatment, preferable in terms of costs, effects and utilities?

a. What are the (extra) costs of the delivery of brief sex counselling compared to intensive sexological treatment and waiting list group?

b. What are the (extra) effects (measured in health status and quality of life) of brief sex counselling compared to intensive sexological treatment and waiting list group?

### Outcome measures

Several instruments will be used to assess the effects of brief sex counselling on sexual functioning, depression and other psychological and physical complaints, and on secondary outcomes. Furthermore instruments will be used to assess the cost-effectiveness of brief sex counselling from a social perspective. The outcomes will be measured before randomization (during initial screening), at baseline and at 3, 6, 9 and 12 months after baseline (Additional file 1).

#### Sexual functioning

##### Questionnaire for the Screening of Sexual Dysfunctions

The Questionnaire for the Screening of Sexual Dysfunctions (QSSD) [29,30] is a self-report instrument with 70 items, each assessed using a 5-point or a 7-point Likert-scale, which aims to measure the presence (and level) of sexual difficulties. This instrument has different versions, dependent on gender, partnered status and gender of the partner. Anchor points used are 'almost never' and 'always' for frequency and 'no trouble' and 'very much trouble' for associated distress.

##### Female Sexual Functional Index

The Female Sexual Functional Index (FSFI) [32] is a multidimensional self-report instrument with 19 items, with responses assessed using a 6-point Likert-scale, which aims to measure sexual functioning on the dimensions of sexual desire, arousal, lubrication, orgasm, satisfaction and pain. High score indicates healthy sexual functioning. Its sound psychometric properties have been proven [35].

##### International Index of Erectile Function

The International Index of Erectile Function (IIEF) [31] is a multidimensional self-report instrument with 15 items, assessed using a 6-point Likert-scale, meant to measure male sexual functioning on the dimensions of erectile functioning, orgasmic functioning, sexual desire, intercourse satisfaction and overall satisfaction. A high score indicates healthy erectile functioning. Its sound psychometric properties have been proven [31].

#### Depression and psychological/physical complaints

##### Center for Epidemiologic Studies Depression Scale

The Center for Epidemiologic Studies Depression Scale (CES-D) [36] is a self-report instrument with 20 items, assessed using a 4-point Likert-scale, meant to measure depressive symptoms on the dimensions somatic-retarded activity, depressed affect, positive affect and interpersonal affect. People with a score of 16 and higher can be identified as "possible cases". Several studies have demonstrated sound psychometric properties [36,37].

##### Brief Symptom Inventory

The Brief Symptom Inventory (BSI) [38] is a self-report instrument to measure psychological distress, with 53 items assessed using a 5-point Likert-scale. The dimensions measured are the same as its predecessor the Symptom Checklist-90. The total score of the BSI can be used as an index of severity for general psychological distress (score range 0-212). Several studies have demonstrated sound psychometric properties [39,40].

##### Medical Outcomes Study 36-item Short Form Health Survey

The Medical Outcomes Study 36-item Short Form Health Survey (SF-36) [41] is a self-report instrument with 36 items, assessed using a 5-point Likert-scale, meant to measure the quality of life. Eight dimensions can be identified: physical functioning, role disabilities caused by physical health problems, pain, general health perception, vitality, social functioning, role disabilities caused by emotional problems, and mental health. These eight dimensions can be summed in a physical and a psychological dimension score. Several studies have demonstrated sound psychometric properties [42,43]. Within the cost-utility analysis, utilities will be derived. The term utility refers to the preferences individuals or society may have for any particular set of health outcomes [44]. The patient's health status will be measured with the SF-36; the Short Form 6D will be used to calculate utilities that incorporate preferences from a general population sample

##### Assessment of DSM-IV Personality Disorders questionnaire

The Assessment of DSM-IV Personality Disorders questionnaire (ADP-IV) [45] is a self-report instrument with 94 items, assessed using a 7-point Likert-scale, meant for a categorical and dimensional measurement of the DSM-IV personality disorders. Several studies have demonstrated sound psychometric properties [46].

#### Economic evaluation

The economic evaluation will be performed from the societal perspective. At the cost side we distinguish three cost-categories: health care sector costs, costs for the patient and family, and productivity costs. For this study we will develop a cost questionnaire especially designed for this group, based on existing questionnaires [25,47], which will identify all relevant cost aspects. To measure the actual use of resources data will be obtained using combined sources (registrations by professionals and cost questionnaire). For the measurement of production losses, the patient modules of the PROductivity and DISease Questionnaire (PRODISQ) will be used [47-49]. Productivity costs will be calculated by means of the friction cost method, based on a mean added value of the Dutch working population. This method takes into account production losses confined to the period needed to replace a sick employee [49].

#### Secondary outcomes

##### Credibility/Expectancy Questionnaire

The Credibility/Expectancy Questionnaire (CEQ) [50] is a multidimensional self-report instrument with 16 items, using on a 9- or 10-point Likert-scale, meant to measure the expectancy and credibility a person has about the therapy received. Its sound psychometric properties have been proven [51].

##### Self-Esteem and Relationship Questionnaire

The Self-Esteem and Relationship Questionnaire (SEAR) [52] is a self-report instrument with 14 items, assessed using a 5-point Likert-scale, meant to measure sexual relationship and psychosocial factors specific for people with sexual problems. Two domains can be distinguished; sexual relationships and confidence. High scores indicate problems with regards to the participant's sexual relationship and confidence. Its sound psychometric properties have been proven [52].

##### Treatment Satisfaction Questionnaire

The Treatment Satisfaction Questionnaire (TSQ) is a new instrument, derived from the Erectile Dysfunction inventory of Treatment Satisfaction (EDITS), and adjusted so it would fit the total study population [53]. This instrument measures the satisfaction of the participants with the treatment as a whole and the different aspects of the treatment. The reliability and validity of de EDITS were well established.

##### Demographic and Biographic Questionnaires

Demographic and biographic questionnaires will be used to assess general information like gender, place of birth, age, occupation, but also the use of medication, drugs and/or alcohol, as well as medical and psychological problems.

### Sample size

In a repeated-measures analysis of the within-between-interaction effect, a sample size of 95 in each group has a power of 95% to detect an effect size of 0.41 using repeated-measures MANOVA with a 0.050 two-sided level of significance. In the absence of published controlled studies on professional care for young people with sexual problems, we set the desired effect size at d = 0.40, representing the central point of the range of effect sizes that are considered as 'medium size' [54]. N = 150 per condition is deemed necessary to guarantee the required number of respondents, in view of the expected non-response after inclusion and later attrition [55]. In a repeated-measures analysis of the within-subjects effect, a sample size of 86 in the treated group has a power of 95% to detect an effect size of 0.25 using a repeated-measures MANOVA with a 0.050 two-sided level of significance. The before mentioned N = 150 per condition is expected to guarantee the required number of participants.

### Randomisation and procedure

For this study a block randomisation procedure will be used to ascertain that each intervention runs in equally large groups. Although the order of the interventions varies randomly within each block, a person running the trial could deduce some of the next treatment allocations if they discovered the block size. In this study we use larger block sizes (30) and randomly vary in block size which can ameliorate this problem [55]. Allocation will be performed by a central independent research assistant after getting informed consent, screening, and checking the in- and exclusion criteria, using Schouten's (1995) adaptive biased urn randomization for small strata. This keeps randomization unpredictable up to and including the last participant on each site, while keeping the group sizes at each site in good balance [56].

The questionnaires will be administered using a computer. All assessments will take place at home via internet. Preceding an upcoming assessment point, participants will receive an email (one week before the end of the treatment and 3 months after the start of the follow-up measurement). If a participant fails to complete the assessment within one week, an email-reminder will be sent. When the participant fails to fill in the questionnaires, a phone call will be made.

### Recruitment

Participants will be recruited through different routes; referrals from Rotterdam-Rijnmond PHS, southern South-Holland PHS, Hollands-middle PHS, Maastricht/Heerlen PHS, Sense, and various websites. Adolescents who visit the PHS for a STI or Sense consult, and who report sexual problems, will be asked if they are interested in participating in the study. Adolescents who visit websites providing sexual information, like http://www.sense.info, can click on a banner if they are interested to participate. After thus reporting their interest, they receive an email containing information about the study protocol, and an informed consent (IC) form. Adolescents who return their signed IC form receive login codes for the questionnaires on the internet. Participants who score above the cut-off score on the QSSD and IIEF/FSFI, and meet the inclusion criteria are included in the study. Participants who do not meet the inclusion criteria are offered suitable alternative treatment options.

### Analyses

#### Clinical analyses

Our primary (base-case) analyses will be performed according to the intention-to-treat principle, including data from all participants regardless of whether they received the intervention or not. For the analyses we will use SPSS statistical software and Excel (for the bootstraps). Respondents for whom at least 75% of the data per measurement instrument are available will be included in the analysis. Missing data on item level will be handled using SPSS missing value analysis. Completely missing measurements will be handled using multiple imputation. Analysis will include comparisons of the intervention groups (brief sex counselling, sexological treatment and waiting list) as well as more complicated multi-level analysis. The accuracy of the findings will be expressed in terms of 95% confidence intervals.

To test the main research questions, difference scores for all outcome variables will be calculated and compared between the three intervention groups using MANOVA. In the case of significant multivariate results, subsequent univariate analysis using independent groups t-tests will be conducted to assess the significance of differences pre/post intervention between control and experimental groups.

#### Economic evaluation

A baseline analysis will be performed to examine the comparability of groups at baseline for both costs and outcomes. If necessary methods will be applied to control for differences in baseline [57]. To investigate whether data are normally distributed a Kolmogorov-Smirnov test will be performed. Despite the usual skewness in the distribution of costs, the arithmetic means will be generally considered the most appropriate measures to describe cost data [58,59]. Therefore arithmetic means (and standard deviations) will be presented. In case of skewness of the cost data, non-parametric bootstrapping will be used to test for statistical differences in costs between the intervention and control group. The bootstrap replications will be used to calculate 95% confidence intervals around the costs (95% CI), based on the 2.5^th ^and 97.5^th ^percentiles. If cost data are distributed normally, t-tests will be used.

The incremental cost-effectiveness ratio (ICER) will be determined on the basis of incremental costs and effects of sex counselling compared to sexological treatment and a waiting-list group. The cost-effectiveness ratio will be stated in terms of costs per outcome rate (increase in sexual functioning, decrease of depressive symptoms) the cost-utility ratio will focus on the net cost per utility gained.

The robustness of the ICER will be checked by non-parametric bootstrapping. Bootstrap simulations will also be conducted in order to quantify the uncertainty around the ICER, yielding information about the joint distribution of cost and effect differences. The bootstrapped cost-effectiveness ratios will be subsequently plotted in a cost-effectiveness plane. The choice of treatment depends on the maximum amount of money that society is prepared to pay for a gain in effectiveness, which is called the ceiling ratio. Therefore, the bootstrapped ICERs will also be depicted in a cost-effectiveness acceptability curve showing the probability that sex counselling is cost-effective using a range of ceiling ratios. Additionally, to demonstrate the robustness of our base-case findings, multi-way sensitivity analyses will be performed [60].

### Collaboration

The current study will be conducted in collaboration with several disciplines. The Rotterdam-Rijnmond PHS will be involved in the development and implementation of brief sex counselling. PsyQ Rotterdam (specialized mental health institute) will be involved in the development and implementation of the intensive sexological treatment. Furthermore, there is collaboration between the Dutch-Flemish research school Experimental Psychopathology (EPP) and the Care and Public Health Research Institute (Caphri).

## Discussion

There are some alarming developments regarding sexual health in adolescents. To overcome the threshold in the treatment of sexual problems, brief sex counselling has been developed. It has been used successively since 2006, but research evaluating this intervention is necessary. Therefore, in the current study we will evaluate the (cost-) effectiveness of brief sex counselling for sexual problems of adolescents and young adults under the age of 25 years of age. We shall compare brief sex counselling with an intensive sexological treatment and a delayed treatment condition.

### Methodological considerations

Brief sex counselling is a relatively new intervention with which the experience in practise is restricted. The intervention is carried out in the - also relatively new - setting of adjuvant sexuality consultations for young people. However, on the basis of the experiences between 2006 and 2008 (pilot version of Sense), the research group is convinced that it is important to start with this intervention because it may reveal sexual health problems that cannot be solved within the regular setting. The threshold for regular sexological treatment might prove to be too high for some groups, who may suffer severe health consequences in later life. Furthermore, this is one of the first economic evaluation studies and the SF-36 does not take into account a sexual dimension. Finally, the questionnaires are administered among a relative young population.

## Conclusion

Brief sex counselling is a new treatment with interesting possibilities. It might lower the threshold to seek professional help for sexual health problems, and could prevent health consequences in later life. The current study contributes to the growing literature on sexual health in adolescents and young adults.

## Competing interests

The authors declare that they have no competing interests.

## Authors' contributions

All authors participated in the design of the study. JJDMvL, SMAAE, EvdV, KvdH obtained funding for the study. AG, JJDMvL and SMAAE drafted the manuscript. All authors read and approved the final manuscript.

## Pre-publication history

The pre-publication history for this paper can be accessed here:

http://www.biomedcentral.com/1471-2458/9/459/prepub

## Supplementary Material

Additional file 1 **Table S1: Instruments used per time point**. Description of the different instruments used at different time points during the study. Click here for file
