# Supplementary material for: Research into the efficacy and cost-effectiveness of brief, free of charge and anonymous sex counselling to improve (mental) health in youth: Design of a randomised controlled trial
Source: BMC Public Health. 2009 Dec 13;9:459. doi: 10.1186/1471-2458-9-459 (PMC2797802; doi:10.1186/1471-2458-9-459)
Supplement: Additional file 1 — Table S1: Instruments used per time point. Description of the different instruments used at different time points during the study. [file 1471-2458-9-459-S1.DOC]

**Table 1: Instruments used per time point**

| **Instrument** | **Screening** | **Baseline** | **3 months** | **6 months** | **9 months** | **12 months** |
| --- | --- | --- | --- | --- | --- | --- |
| **QSSD**  **FSFI**  **IIEF**  **CEQ**  **BSI**  **CES-D**  **SEAR**  **SF-36**  **TSQ**  **D/BQ**  **ADAP-IV**  **CQ** | **X**  **X**  **X**  **X**  **X**  **X**  **X**  **X** | **X**  **X**  **X**  **X**  **X**  **X**  **X**  **X**  **X** | **X**  **X**  **X** | **X**  **X**  **X**  **X**  **X**  **X**  **X**  **X**  **X** | **X**  **X**  **X** | **X**  **X**  **X**  **X**  **X**  **X**  **X**  **X** |

QSSD= Questionnaire for the Screening of Sexual Dysfunction

FSFI= Female Sexual Functional Index

IIEF= International Index of Erectile Function

CEQ= Credibility/expectancy Questionnaire

BSI= Brief Symptom Inventory

CES-D= Center for Epidemiologic Studies Depression Scale

SEAR= Self-Esteem and Relationship Questionnaire

SF- 36= Medical Outcomes Study 36-item Short Form Health Survey

TSQ= Treatment Satisfaction Questionnaire

D/BQ= Demographic and biographic questionnaires

ADAP-IV= Assessment of DSM-IV Personality Disorders questionnaire

CQ= Cost questionnaire
